# Exploring the role of the MUC1 mucin in human oral lubrication by tribological in vitro studies

**DOI:** 10.1038/s41598-024-82176-2

**Published:** 2024-12-28

**Authors:** Ianis Ammam, Cyril Pailler-Mattéi, Lucas Ouillon, Clément Nivet, Roberto Vargiolu, Fabrice Neiers, Francis Canon, Hassan Zahouani

**Affiliations:** 1 Ecole Centrale de Lyon, CNRS, ENTPE, LTDS, Ecully, UMR5513, 69130 France; 2 Centre des Sciences du Goût et de l’Alimentation, UMR1324 INRAE, UMR6265 CNRS Université de Bourgogne, Institut Agro Dijon, Dijon, F-21000 France; 3 Guy de Collongue, Laboratoire de Tribologie et Dynamique des Systèmes, Ecole Centrale de Lyon, Université de Lyon, UMR-CNRS 5513, Ecully, 69134 France

**Keywords:** Biomaterials - cells, Biomaterials, Tissues, Mechanical engineering

## Abstract

In the context of the oral cavity, an organic layer known as the mucosal pellicle (MP) adheres to the surface of the oral epithelium, playing a pivotal role in lubricating and safeguarding oral tissues. The formation of the MP is driven by interactions between a transmembrane mucin known as MUC1, located on the oral epithelium, and salivary secreted mucin, namely MUC5B and MUC7. This study aimed to investigate the function of MUC1 and the influence of its structure on MP lubrication properties. We proposed a novel methodology to study oral lubrication based on four different models of oral epithelium on which we conducted in vitro tribological studies. These models expressed varying forms of MUC1, each possessing on of the distinct domain constituting the mucin. Mechanical parameters were used as indicators of lubrication efficiency and, consequently, of the role played by MUC1 in oral lubrication. The results from the tribological tests revealed that the presence of full MUC1 resulted in enhanced lubrication. Furthermore, the structure of MUC1 protein drive the lubrication. In conclusion, the mechanical tests conducted on our epithelium models demonstrated that MUC1 actively participates in epithelium lubrication by facilitating the formation of the MP.

## Introduction

The oral cavity supports various essential functions for organisms, encompassing food processing (ingestion, mastication, and bolus formation), flavour perception, speech, defence, and respiration. Notably, processes such as food oral processing and speaking subject the oral mucosa to mechanical forces including shear and friction, arising from activities like chewing and speaking, potentially causing damage. In response to these stresses, saliva, a lubricating fluid, is continuously secreted in the oral cavity, and participates in the formation of a protective layer, bound at the surface to the oral mucosa, known as the mucosal pellicle (MP).

The MP is mainly composed of salivary proteins specifically anchored at the surface of the oral epithelium, forming a mucus^1^. The main organic components of mucus are the gel-forming mucins, enabling reversible interactions that drive the formation of the mucus gel^2^. Its composition has been characterised through in vivo and in vitro studies which have identified specific salivary mucins MUC5B and MUC7, as well as proteins rich in prolines (PRPs) and immunoglobulin A (IgA)^1,3–7^. Furthermore, these investigations have unveiled the presence of a transmembrane mucin, MUC1, expressed by epithelial cells.

Despite its significance, understanding of the mechanism behind MP formation remains incomplete. Recent findings suggest that molecular interactions involving the epithelial mucin MUC1 and salivary mucins MUC5B and MUC7^8 ^play a major role in MP formation. Studies by Ployon et al. and Aybeke et al. underscored the crucial role of MUC1 in facilitating the anchoring of salivary proteins to the oral epithelium model, utilizing the TR146 cell line^9,10^. This is consistent with previous research reporting the implication of MUC1 in MP formation^11,12^. Although the precise nature of the molecular interactions remains elusive, hydrophobic effects appear to be involved^13^, linking the hydrophobic domains of MUC1 and MUC5B. MUC1 is a multifaceted protein comprised of two subunits (α and β) intricately connected by non-covalent bonds^14,15^. The α-subunit, located entirely extracellularly, is predominantly composed of a highly glycosylated variable number tandem repeat (VNTR) domain^16^. This subunit encompasses the N-terminal part, that accommodates the binding site with MUC5B, along with a segment of a domain called the SEA domain. In contrast, the β-subunit comprises a concise extracellular domain, encompassing a portion of the SEA domain, a transmembrane domain, and the intracellular section of the protein. The cytoplasmic tail contains signaling motifs and seven phosphorylation sites, substantiating MUC1’s role as a signaling protein. Originally derived from the same pro-protein, the two subunits undergo the hydrolysis of a cleavage site within the SEA domain, resulting in the formation of two subunits that remain bound together through non-covalent interactions. Lindèn et al. proposed that the dissociation of these subunits may constitute a defence mechanism against epithelial aggressions^17^. This dissociation could potentially trigger the phosphorylation of an MUC1 cytoplasmic tail, activating an intracellular pathway^14^. Beyond its signalling function, MUC1 also plays a pivotal role in the lubrication of the epithelial surface owing to its glycosylation, thereby safeguarding the oral mucosa^10,16,18,19^. Some studies have shown that MUC1 plays a role in tissue lubrication in general, as observed through the study of oral tribology^18,20–22^.

Numerous studies have demonstrated that tribology, the science of friction, lubrication, and wear, provides a pertinent approach for investigating the intricate mechanisms and interactions involved in oral processes, particularly the lubricating properties of saliva^23–28^. The oral process is inherently dynamic, involving complex movements between different surfaces such as the palate, tongue, teeth and oral mucosa, resulting in frictional forces between these components. Many studies have used tribology to characterise oral lubrication, employing various materials to mimic oral tissues (PDMS, ex vivo porcine tongue, hydrogel, etc.)^23–25,29–32^. However, the majority of these studies used synthetic or ex vivo materials, posing challenges in studying the interaction between salivary proteins and proteins involved in forming the mucosal pellicle.

In this study, we build on the hypotheses proposed in the literature that MUC1 plays a crucial role in promoting oral lubrication through the formation of MP^11,12,14^. The aim of this work is to validate these hypotheses through in vitro tribological assays, specifically focusing on three aspects: i, MP is an efficient lubricating layer; ii, MUC1 participates in the lubricating properties of the mucosal pellicle; iii, examining the role of MUC1’s structure, with a specific focus on the VNTR and SEA domains, within the context of the lubrication process. To achieve these goals, we conducted in vitro tribological studies utilizing oral epithelium models expressing MUC1 ^10,33^. These epithelium models are composed of the TR146 cell line expressing or not different isoforms of MUC1, along with a reconstituted PM after the incubation of TR146 cells with saliva. Mechanical parameters, including the friction coefficient ($\:\mu\:)$, energy dissipated by friction ($\:Ed$), and damage surface, were measured from the tribological tests and served as indicators of the state of lubrication and, consequently, the role played by MUC1 in oral lubrication. The tribological tests were conducted using a device developed in this study.

## Results and discussion

### Functions of MUC1 in the lubrication of the epithelium model

**Fig. 1:**
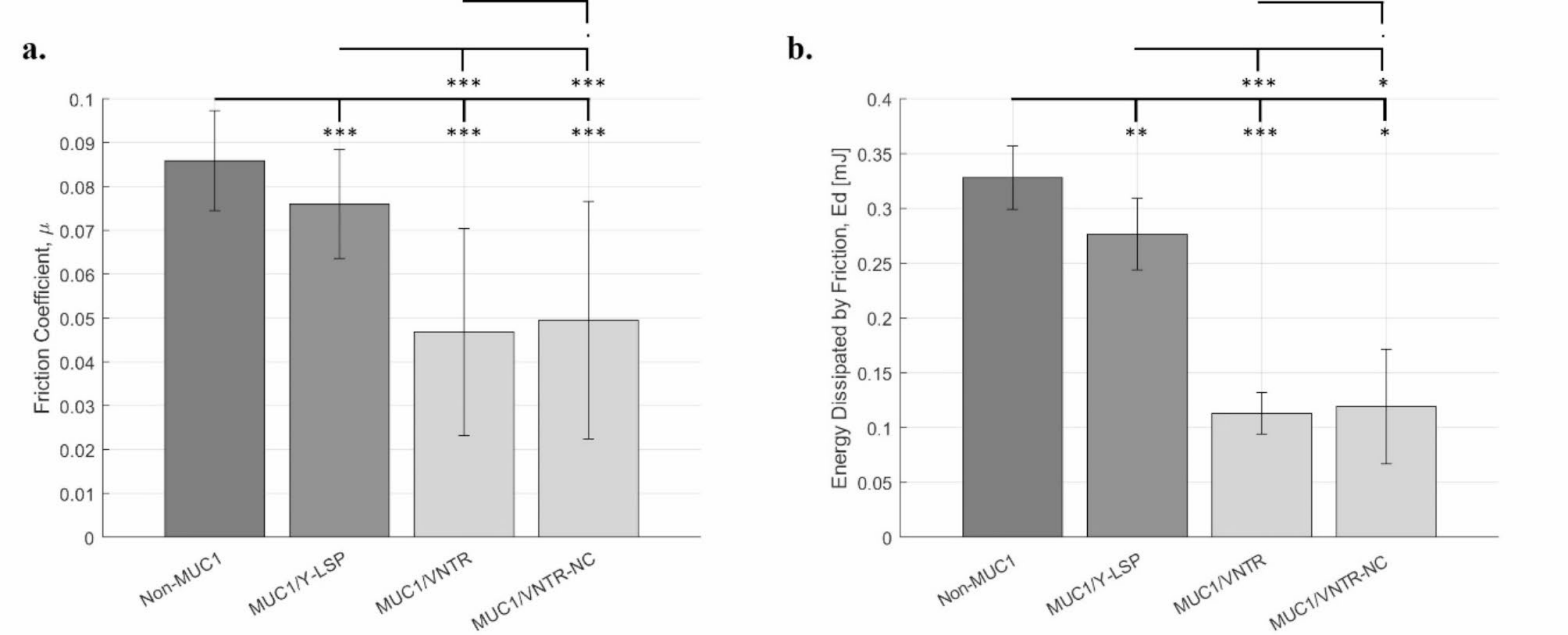
(**a**) Friction coefficient and (**b**) Energy Dissipated on the four isoforms of MUC1 without reconstruction of the MP (mean values (*n* = 12) +/- standard deviation). Statistical results were obtained using the method described in Sect. 3.5 - Signification codes: 0 < “***” < 0.001 < “**” < 0.01 < “*” < 0.05 < “.”.

One of the crucial functions of MUC1 is the lubrication of epithelium, especially in the oral cavity^34,35^. Before examining the role of this mucin in the lubricating properties of the MP under physiological conditions, the impact of MUC1 and its structure on the lubrication of epithelium models without reconstructing the MP was investigated. Figure 1 shows the friction forces and the energy dissipated in these conditions depending on the cell lines.

MUC1 contributes to the lubrication of surfaces where it is expressed, located at the apical surface of epithelial cells, forming a gel that lubricates tissues^36^. Yakubov et al., confirmed that an increase in mucin concentration leads to a reduction in frictional forces during friction tests^37^. Other research has examined and demonstrated the lubricating effects of gel-forming mucin in a tribological context^29,38^; however, MUC1 is a tethered mucin. Our research also revealed that the absence of MUC1 expression in epithelial models results in an increase in frictional forces compared to isoforms expressing MUC1. Indeed, the evaluation of friction coefficient and energy dissipated (Fig. 1) supports these results. Parameters decreased in presence of MUC1 (*p* < 0.001, see Table 1). The friction coefficients were $\:0.086\pm\:0.011$ for the non-MUC1 isoform and $\:0.076\pm\:0.012$, $\:0.047\pm\:0.023$ and $\:0.050\pm\:0.027$ for cell lines expressing MUC1 (MUC1/Y-LSP, MUC1/VNTR and MUC1/VNTR-NC, respectively).

MUC1, as a tethered mucin, is implicated in the lubrication of the surface of epithelial cells^39^. The heavily glycosylated VNTR of MUC1 may potentially participate in the retention of water molecules and the formation of a mucous gel^39^, promoting an optimal hydrophilic environment for the hydration and lubrication of epithelia^40,41^. Thus, variations in lubrication at the epithelial cell surface were expected based on the structure of MUC1, especially the presence of the VNTR domain. By enhancing lubrication^41^, the VNTR domain protects the underlying epithelium. Figure 1 depicts these findings (*p* < 0.001, Table 1). The MUC1/Y-LSP isoform, which lacks the VNTR domain and thus glycosylation, exhibits less effective lubrication ($\:Ed=\:0.28\pm\:0.033 mJ)\:$ than the cell lines composed of a heavily glycosylated VNTR domain: MUC1/VNTR and MUC1/VNTR-NC ($\:Ed=\:0.11\pm\:0.02mJ$ and $\:Ed=\:0.12\pm\:0.05 mJ\:\:$respectively).

Our study reveals that the presence of this SEA domain results in a minimal decrease in frictional forces ($\:\mu\:$ and $\:Ed)$ (Fig. 1) on the epithelial surfaces compared to the frictional forces observed between MUC1/VNTR isoforms (composed of a VNTR and SEA domain) and MUC1/VNTR-NC (with a non-cleavable SEA domain). These decreases are not significant (Table 1) for either parameter.

**Table 1:** P-values obtained from statistical tests based on the analysis presented in Sect. 3.5. These tests compare the effect of MUC1 presence without MP on the friction coefficient and the energy dissipated. Signification codes: 0 < “***” < 0.001 < “**” < 0.01 < “*” < 0.05 < “.“.

Isoform	Isoform	Friction coefficient	Energy Dissipated
		$\:{p}_{value}$	$\:{p}_{value}$
Non-MUC1	MUC1/Y-LSP	$\:0.00015$	$\:0.008$
Non-MUC1	MUC1/VNTR	$\:{6.5\cdot\:10}^{-20}$	$\:2.2\cdot\:{10}^{22}\:$
Non-MUC1	MUC1/VNTR-NC	$\:2.3\cdot\:{10}^{-12}$	$\:0.02$
MUC1/Y-LSP	MUC1/VNTR	$\:9.9\cdot\:{10}^{-11}$	$\:2.8\cdot\:{10}^{-19}\:$
MUC1/Y-LSP	MUC1/VNTR-NC	$\:2.6\cdot\:{10}^{-6}$	$\:0.01$
MUC1/VNTR	MUC1/VNTR-NC	$\:0.9$	$\:0.3$

### Effects of MUC1 in influencing the lubricating properties of the mucosal pellicle

**Fig. 2:**
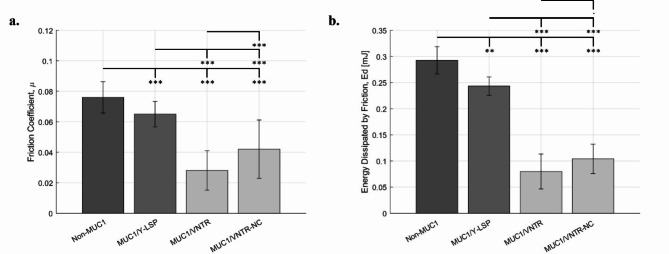
(**a**) Friction coefficient and (**b**) Energy Dissipated obtained after the formation of the MP on oral epithelium models for each MUC1 isoform (mean values (*n* = 12) +/- standard deviation). Statistical results were obtained using the method described in Sect. 3.5 - Signification codes: 0 < “***” < 0.001 < “**” < 0.01 < “*” < 0.05 < “.”.

**Fig. 3:**
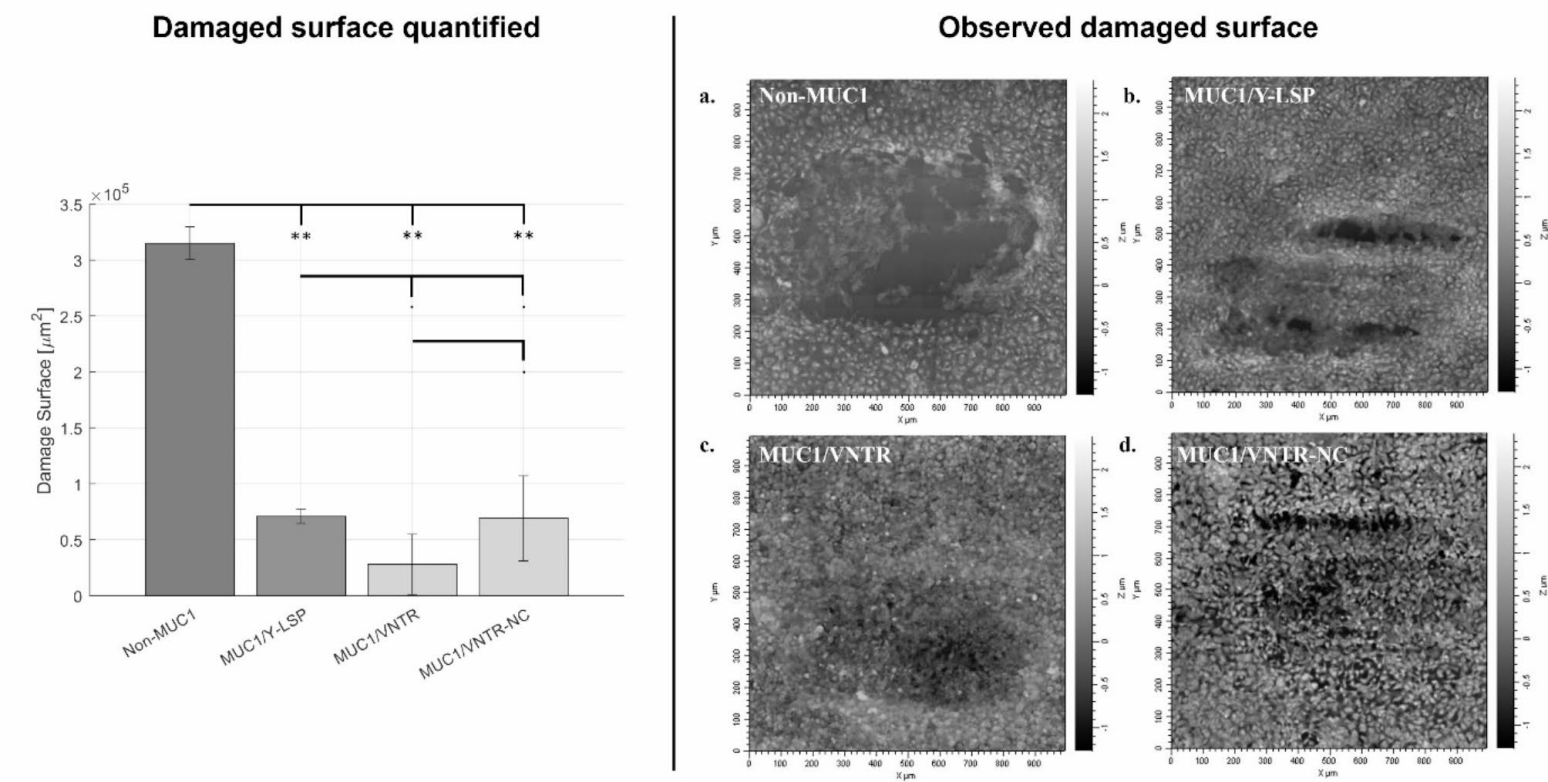
Figure showing surface damage induced by friction for the four MUC1 isoforms after MP reconstruction. Surface damage is presented both quantitatively and visually - On the left, bar charts display the quantified damage for each isoform (mean values (*n* = 12) ± standard deviation). Statistical results were obtained using the method described in Sect. 3.5 - The images on the right show the damage observed through interferometry, providing qualitative analysis. Signification codes: 0 < “***” < 0.001 < “**” < 0.01 < “*” < 0.05 < “.”.

Our friction test results, exploring three mechanical parameters, offer insights into the lubrication state of our oral epithelium models. Notably, Fig. 2 reveals a significant reduction in the friction coefficient and energy dissipation (*p* < 0.001, refer to Table 2) for the (MP) formed in the presence of MUC1. This prompts the hypothesis that MUC1 and MUC5B contribute collaboratively to lubricating the surface of the oral mucosa. Figure 2 shows the friction forces and the energy dissipated depending on cell lines, while Fig. 3 presents the damage surface after friction. These parameters are indicators of the lubrication state of the oral epithelium models.

The friction coefficient and the energy dissipated decreased significantly (*p* < 0.001, see Table 2) in the presence of MUC1, confirming its role in promoting lubrication in our oral epithelium model. For the TR146 cell line (without MUC1), the friction coefficient ($\:\mu\:)$ and dissipated energy ($\:Ed)$ were $\:0.076\pm\:0.014$ and $\:0.29\pm\:0.026\:\text{m}\text{J}\:,\:\text{r}\text{e}\text{s}\text{p}\text{e}\text{c}\text{t}\text{i}\text{v}\text{e}\text{l}\text{y}$. In contrast, for MUC1 isoforms (MUC1/Y-LSP, MUC1/VNTR, MUC1/VNTR-NC), the values were all lower: $\:\left(0.065\pm\:0.011\right),\left(\:0.028\:\pm\:0.013\right),\text{a}\text{n}\text{d}\:(\:0.042\pm\:0.0.23)\:\:$for $\:\mu\:$ and $\:\left(0.24\pm\:0.017\right)mJ,\:\left(0.079\pm\:0.033\right)mJ,\:\text{a}\text{n}\text{d}\:\left(0.10\pm\:0.028\right)mJ$ for $\:Ed,\:\text{r}\text{e}\text{s}\text{p}\text{e}\text{c}\text{t}\text{i}\text{v}\text{e}\text{l}\text{y}$. These lower values indicate that the presence of MUC1 enhances lubrication, resulting in reduced friction forces.

Furthermore, the evaluation of damage surface after friction (Fig. 3) supports these findings. The images reveal more significant deteriorations for the TR146 cell line (non-MUC1) than for the TR146 cell lines expressing isoforms of MUC1. Quantitatively, the deterioration surface decreases in the presence of MUC1 ($\:p<0.01$, see Table 2). More specifically, the surface for the TR146 cell line is $\:(3.15\pm\:0.15)\cdot\:{10}^{5}\mu\:{m}^{2}$, whereas for the TR146 cell line expressing MUC1 isoforms, it is $\:\left(0.71\pm\:0.062\right)\cdot\:{10}^{5},\:\left(0.28\pm\:0.268\right)\cdot\:{10}^{5}\mu\:{m}^{2},\:and\:\:\left(0.69\pm\:0.38\:\right)\cdot\:{10}^{5}\mu\:{m}^{2}$, respectively. This discrepancy highlights the impact of MUC1 on reducing damage, emphasizing its role in enhancing the protective function of the mucosal pellicle.

Our results reveal notable distinctions in the lubricating properties of the MP across the different cell lines, with the weakest lubrication observed in the parental cell line (non-expressing MUC1). Importantly, MUC1 enhances the anchoring of salivary proteins^9,10,33^and modulates the composition^1^, physico-chemical properties^9^, and structure of the MP, thereby influencing tribological dynamics. More specifically, MUC1 has been reported to increase the binding of salivary proteins, especially the gel forming mucin MUC5B^10–12,33^, modulating the lubricating properties of the MP. This increased anchoring of MUC5B at the epithelial cell surface, facilitated by MUC1, likely contributes to the augmentation observed of the lubricating properties of the MP^5,42–45^. Our results support the hypothesis of Chang et al., who suggested that a decrease in MUC1 expression may lead to reduced oral mucosal defences, possibly due to decreased lubrication^46^. Our findings agree with the insights presented by Mu and Chen in their review^47^ underscoring that interactions between MUC1 and other salivary mucins could result in the establishment of an initial lubricating layer.

Furthermore, MUC1, as a tethered mucin, is implicated in the lubrication of the surface of epithelial cells^39^. The heavily glycosylated VNTR of MUC1 may potentially participate in the retention of water molecules and the formation of a mucous gel^39^. Thus, variations in lubrication at the epithelial cell surface are expected based on the structure of MUC1, especially the presence of the VNTR domain.

**Table 2:** P-values obtained from statistical tests based on the analysis presented in Sect. 3.5. These tests compare the effect of MUC1 on the MP lubrication properties, on the friction coefficient, energy dissipated, and surface damage. Significance codes: 0 < “***” < 0.001 < “**” < 0.01 < “*” < 0.05 < “.” .

Isoform	Isoform	Friction coefficient	Energy dissipated	Damage surface
		$\:{p}_{value}$	$\:{p}_{value}$	$\:{p}_{value}$
Non-MUC1	MUC1/Y-LSP	$\:0.00012$	$\:0.003$	$\:0.008$
Non-MUC1	MUC1/VNTR	$\:1.2\cdot\:{10}^{-20}$	$\:0.00054$	$\:0.008$
Non-MUC1	MUC1/VNTR-NC	$\:8.2\cdot\:{10}^{-18}$	$\:0.00084$	$\:0.005$

In conclusion, the comparison of mechanical parameters, whether with (Fig. 2) or without (Fig. 1) MP reconstruction, shows a significant decrease after reconstruction of the MP. This supports the lubricating power of the MP. Table 3 indicates the rate of variation of the friction coefficient and the energy dissipated ($\:{\Delta\:}\mu\:,\:{\Delta\:}Ed$ respectively) between both conditions: with and without MP reconstruction. This table delineates the lubricating properties of the MP contingent upon the variation of MUC1 isoforms. Both $\:{\Delta\:}\mu\:$ and $\:{\Delta\:}Ed\:$demonstrate significant negative values across all cell lines ($\:{\Delta\:}\mu\:<0,\:{\Delta\:}Ed<0$), signifying a reduction in friction forces at the oral epithelial cell surface following the specific anchoring of salivary proteins forming the MP. The result attained unequivocally establishes that the MP significantly impacts the reduction of friction forces at the surface of the epithelial oral cells, thereby confirming our initial hypothesis concerning its lubricating role in the oral epithelium. These findings are in line with earlier research. Previous tribological investigations on synthetic surfaces (PDMS, Agarose) have similarly explored the lubricating attributes of the MP, reporting a decrease in the friction coefficient^24,48^.

Furthermore, variations were discernible between the different cell lines. The lowest negative values for $\:{\Delta\:}\mu\:$ and $\:{\Delta\:}Ed$ were observed for the parental cell line (TR146), which lacked MUC1, with values of −11.7% and − 10.8%, respectively. The second-lowest values were recorded for MUC1/Y-LSP, expressing a short isoform of MUC1 without the VNTR domain and with a non-cleavable SEA domain ($\:\:{\Delta\:}\mu\:=\:$−14.6% and $\:{\Delta\:}Ed=$ −12%). The most pronounced negative values were obtained for the TR146-MUC1/VNTR cell line (−40.4% and − 29.4%), encompassing the VNTR domain and a cleavable SEA domain. In contrast, its closed-pending isoform MUC1/VNTR-NC, distinguished by a non-cleavable SEA domain, exhibited the second highest negative values (−15.4% and − 12.7% respectively). These results underscore the influence of the MUC1 structure on the lubricating properties of the MP.

**Table 3:** Rate of variation of: Friction Coefficient and Energy dissipated for each cell line between both conditions: without and with MP reconstruction.

Isoforms	Friction coefficient variation rate$\:{\Delta\:}\mu\:$(%)		Energy dissipated variation rate$\:{\Delta\:}Ed$(%)
Non-MUC1	$\:-11.7$		$\:-10.8$
MUC1/Y-LSP	$\:-14.6$		$\:-12.0$
MUC1/VNTR	$\:-40.4$		$\:-29.4$
MUC1/VNTR-NC	$\:-15.4$		$\:-12.7$

### Effect of the MUC1 structure

#### Study of the function of the VNTR domain in the formation of MP

This study marks a ground-breaking exploration into the influence of the structure of MUC1 on the lubricating properties of the MP. Notably, our findings shed light on the differential effect among MUC1 isoforms, with the weakest impact observed with MUC1/Y-LSP, which lacks the highly glycosylated VNTR domain. In contrast, isoforms incorporating VNTR exhibited the most pronounced effect. Figure 2 depicts the friction forces and energy dissipated based on cell lines, while Fig. 3 also illustrates the damage surface after friction.

Table 4 highlights the significance of differences between MUC1/Y-LSP and both MUC1/VNTR and MUC1/VNTR-NC. The friction coefficient for the TR146-MUC1/Y-LSP cell line is approximately $\:(0.065\pm\:0.011)$, while for the two cell lines expressing MUC1 with the VNTR domain (TR146-MUC1/VNTR and TR146-MUC1/VNTR-NC) the coefficients are$\:\:(0.028\:\pm\:0.013)$ and $\:(0.042\pm\:0.0.23)$ respectively. These differences are highly significant. In terms of dissipated energy, a similar trend supporting the energy dissipation of the SEA domain in the presence of the salivary proteins forming the MP, is observed: TR146-MUC/Y-LSP exhibits a calculated $\:Ed$ of $\:\left(0.24\pm\:0.017\right)mJ$, whereas for the cell line expressing MUC1 isoforms with the VNTR domain, $\:Ed$ is $\:\left(0.079\pm\:0.033\right)mJ$ and $\:\left(0.10\pm\:0.028\right)mJ$ (MUC1/VNTR and MUC1/VNTR-NC respectively). These distinctions are statistically significant, as indicated in Table 4.

Concerning the damage surface after friction, qualitative analysis of wear marks in Fig. 3 indicates that friction on TR146-MUC1/Y-LSP cell lines (without a VNTR domain) causes more degradation than on the cell line expressing MUC1 isoforms with a VNTR domain (MUC1/VNTR and MUC1/VNTR-NC). However, the protective effect of a cleavable SEA-domain is not observable in the condition tested probably due to the slight damage observed in the presence of the VNTR domain. Indeed, the surface area damaged by friction is very small and nearly identical for the MUC1/Y-LSP and MUC1/VNTR-NC isoforms. The degradation area for MUC1/Y-LSP is $\:\left(0.71\pm\:0.06\right)\cdot\:{10}^{5}\mu\:{m}^{2}$. For isoforms presenting the VNTR domain (MUC1/VNTR and MUC1/VNTR-NC), the surfaces were $\:\left(0.28\pm\:0.268\right)\cdot\:{10}^{5}\mu\:{m}^{2}$ and $\:\left(0.69\pm\:0.38\right)\cdot\:{10}^{5}\mu\:{m}^{2}$ respectively. However, there was a significant reduction in the damaged surface area for the MUC1/VNTR isoform compared to the other two, without the VNTR domain or without the cleavable SEA domain.

This suggests that the VNTR domain contributes to the lubricating properties of the MP either independently or synergistically with MUC5B and MUC7.

The highly glycosylated nature of the VNTR domain, which confers lubricating properties to MUC1 ^16^, is significant. The negative charge of the VNTR domain may participate in the formation of the mucosal gel with MUC5B through interaction and water molecule retention. These findings are in line with the existing literature, emphasizing the role of the VNTR domain in the lubrication of epithelial cells. For instance, Bodega et al. observed an elevated friction coefficient in tumour cells, possibly due to the expression of MUC1, disrupting its lubricating properties^20^. Another study supported the role of VNTR in lubrication, demonstrating that in corneal epithelial cells, the glycosylation domain contributes to the anti-adhesive properties of mucin, and that alterations in this domain lead to mucosal dryness, a condition characterised by increased friction^18^.

**Table 4:** P-values obtained from statistical tests based on the analysis presented in Sect. 3.5. These tests compare the effect of VNTR domain on MP lubrication properties, on the friction coefficient, energy dissipated, and surface damage. Significance codes: 0 < “***” < 0.001 < “**” < 0.01 < “*” < 0.05 < “.” .

Isoform	Isoform	Friction coefficient	Energy dissipated	Damage surface
		$\:{p}_{value}$	$\:{p}_{value}$	$\:{p}_{value}$
MUC1/Y-LSP	MUC1/VNTR	$\:2.23\cdot\:{10}^{-9\:}$	$\:1.22\cdot\:{10}^{-5\:}\:$	$\:0.09$
MUC1/Y-LSP	MUC1/VNTR-NC	$\:1.37\cdot\:{10}^{-7}$	$\:0.0002$	$\:0.27$

#### The involvement of the SEA domain in MP formation on the Epithelium Model

In our comprehensive investigation employing consistent mechanical parameters, we performed a detailed examination of the pivotal role played by the SEA cleavable domain in regulating lubrication dynamics in our oral epithelium models. To achieve this, we conducted a comparative analysis between the two distinct MUC1 isoforms: MUC1/VNTR and the MUC1/VNTR-NC. These two isoforms of MUC1 differ by a 15-amino-acid deletion, corresponding to the following amino acid sequence “GVSFFFLSFHISNLQ”. It is noteworthy that maintaining an intact protein structure is crucial for the proper folding of a cleavage-competent SEA domain structure, as described by Levitin et al.^15^. This 15-amino-acid deletion impedes the cleavable function of the SEA domain^15^. Both MUC1/20VNTR-NC and MUC1/Y-LSP share this 15-amino-acid deletion, forming a monomeric structure compared to the heterodimeric structure of the cleavable SEA domain-containing structure.

Our findings illustrated in Figs. 2 and 6, clearly demonstrate a significant reduction in friction forces and damage levels in the presence of a two-subunit MUC1, attributable to the cleavable SEA domain (see Table 5). With regards to energy dissipation $\:Ed$ and damage surface, the decrease observed does not reach statistical significance at a p-value of 0.05. This reduction in friction force and degradation surface in the presence of a two-subunit MUC1 may be attributed to the disruption of noncovalent forces that bond the two subunits together under mechanical stress. This disruption likely contributed to the dissipation of friction forces during the mechanical assay, as previously suggested by Levitin et al.^15^.

It has been proposed that the self-cleaving SEA domain has evolved to undergo dissociation in response to mechanical stress rather than chemical stress at the apical cell membrane. This mechanism serves as a protective measure to prevent cell rupture, as discussed by Macao et al.^49^. Furthermore, it has also been suggested that the cell can detect mechanical shear at the mucosal surface if dissociation is signalled through cellular signalling following the loss of a SEA-binding protein, as also proposed by Macao et al.^49^. This mechanism may play a role in the astringency sensation during the aggregation of the mucosal pellicle by tannins, as proposed by Canon et al. (Canon et al., 2021). Our results unequivocally validate the efficiency of this mechanism in dissipating friction forces effectively under mechanical stress at the cell surface, thereby minimizing cell damage. Indeed, the isoform containing the cleavable SEA domain consistently exhibits the lowest level of damage, coupled with the weaker friction forces on its surface, in line with our scientific understanding.

**Table 5:** P-values obtained from statistical tests based on the analysis presented in Sect. 3.5. These tests compare the effect of SEA domain on MP lubrication properties, on the friction coefficient, energy dissipated, and surface damage. Significance codes: 0 < “***” < 0.001 < “**” < 0.01 < “*” < 0.05 < “.” .

Isoform	Isoform	Friction coefficient	Energy dissipated	Damage Surface
		$\:{p}_{value}$	$\:{p}_{value}$	$\:{p}_{value}$
MUC1/VNTR	MUC1/VNTR-NC	$\:7\cdot\:{10}^{-7}$	$\:0.07$	0.13

## Materials and methods

Tribological experimental tests were conducted on an in vitro model of the oral epithelium, based on the TR146 cell line with a reconstituted MP. Four different TR146 cell lines were used. Three of these cell lines were derived from a parental TR146 cell line that does not express MUC1 and which was transfected by three different genes coding for three MUC1 isoforms. Tribological measurements were conducted on each cell line with or without MP. From these measurements, mechanical parameters such as friction coefficient and energy dissipated by friction were calculated. Images of the friction trace at the epithelium surface were acquired for each condition to calculate the damage surface. These mechanical parameters were used as indicators of the surface lubricating properties depending both on MUC1 isoforms and the presence of the MP.

This section describes in detail the experimental protocols used to prepare the samples and the tools used to carry out the mechanical tests.

### Saliva Collection

The study was conducted in accordance with the Declaration of Helsinki guidelines, it received approval from the Ethics Committee for Research (CPP Est I. Dijon, #14.06.03, ANSM #2014-A00071-46). Saliva was collected from healthy individuals who provided their written informed consent. The donors did not drink, smoke or eat for 2 h prior to collection, and spat out into plastic containers the saliva that accumulated naturally in their mouths, thus providing unstimulated saliva. The saliva was used immediately or frozen immediately in liquid nitrogen to avoid degradation of the biological material.

### In vitro epithelium model

#### Four different cell lines

**Fig. 4:**
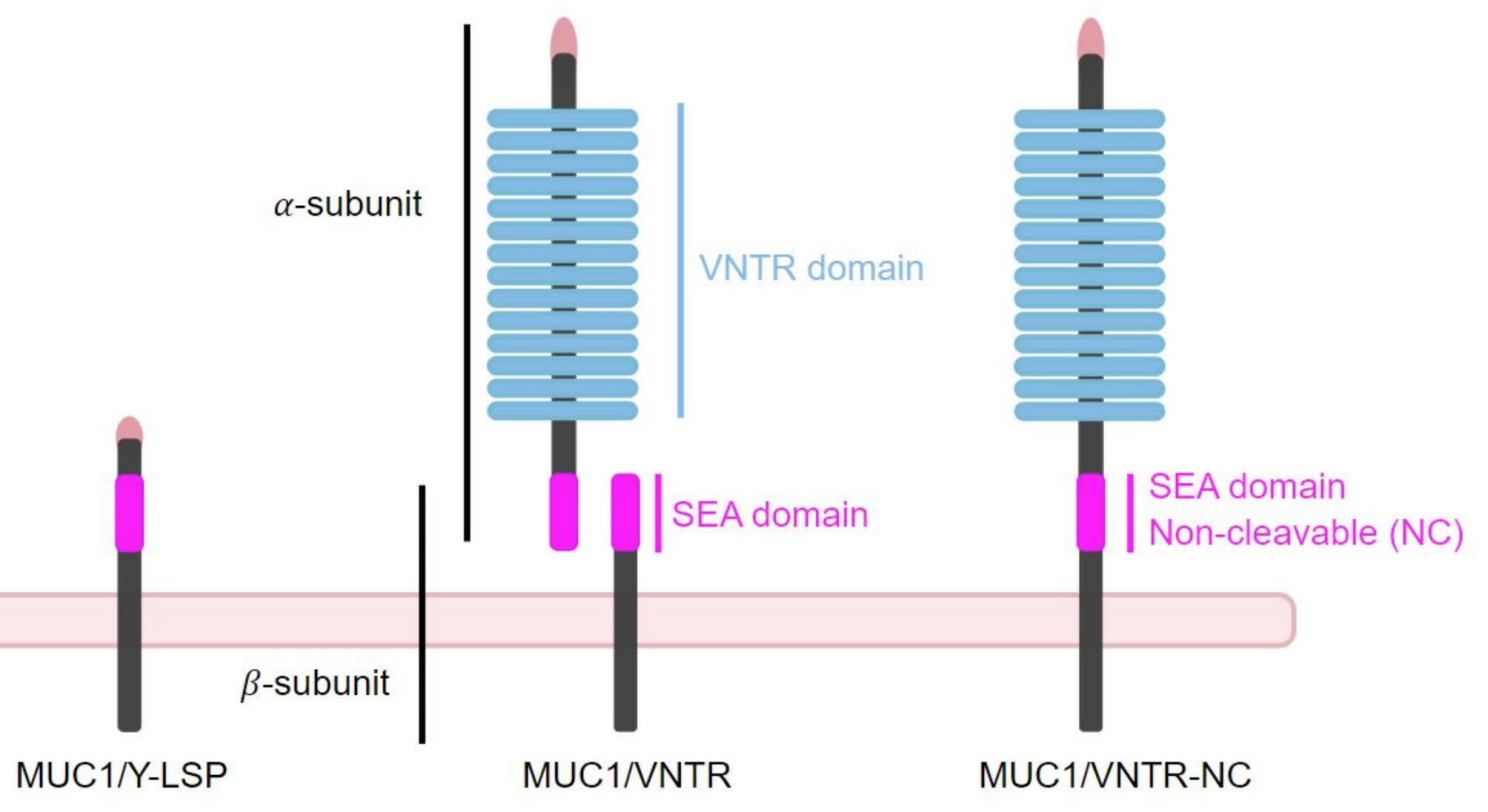
Diagram of the 4 MUC1 isoforms studied in this work. Non-MUC1 does not express the MUC1 protein. MUC1/Y-LSP expresses a truncated form of MUC1, which is shorter. MUC1/VNTR expresses a form of MUC1 with the VNTR domain and the cleavable SEA domain. MUC1/VNTR-NC expresses MUC1 with the VNTR domain but without the SEA domain.

As explained above, each of the 3 cell lines derived from the TR146 parental cell line, which does not express MUC1, were transfected with a gene coding for a different isoform of MUC1. These cell lines were developed and supplied to us by the CSGA (Centre des Sciences du Goût et de l’Alimentation, Dijon, France) as part of our research project. The 4 cell lines studied were characterized previously^10,33^ and named depending on the MUC1 isoforms they express: non-MUC1, MUC1/Y-LSP, MUC1/VNTR, MUC1/VNTR-NC. Figure 4 presents the structural characteristics of MUC1 isoforms depending on the cell line. The non-MUC1 cell line (TR146) lacks expression of MUC1. The TR146-MUC1/Y-LSP cell line expresses an MUC1 isoform lacking the VNTR domain and having a truncated SEA domain. The TR146-MUC1/VNTR encodes an isoform of MUC1, close to the wild isoforms, with 2 subunits $\:\alpha\:$ and $\:\beta\:$, the VNTR domain and a full SEA domain (cleavable). The TR146-MUC1/VNTR-NC cell line expresses an isoform similar to MUC1/VNTR but constituted by only one subunit as it contains a truncated SEA domain, which precludes the cleavage of MUC1 into two subunits.

#### Cell culture

Cells were routinely cultured in Dmem/F12 + GlutaMax (1:1, v: v) supplemented with 10% foetal bovine serum (FBS) and 100units/ml penicillin, 100 mg/ml streptomycin in T75 flasks (all from Life Technologie, Germany). For the MUC1/Y-LSP isoform, the medium was supplemented with 0.2% geniticin. Regarding isoforms MUC1/VNTR and MUC1/VNTR-NC we added 0.025% zeocin. Cells were subcultured every 4 days and the medium was changed every 2 days. Cells were incubated at 37 °C in a humidified atmosphere containing 7.5% CO_2_. Cells were seeded at a density of $\:0.75\cdot\:{10}^{6}\frac{cells}{mL}$ onto glass slides which acted as the substrate in 24-well plates. The glass slides were previously coated with Poly-D-Lysine (Gibco, TermoFischer, Germany). Confluence was reached in 24 h.

The mucosal pellicle was reconstituted at the cell surface after incubation of the cell monolayer with saliva for 2 h. After 2 h, the cells were rinsed with DPBS (TermoFischer, Germany) twice to remove saliva that had not adhered to the epithelium, leaving only the MP anchored to the mucosa model. The in vitro epithelium model consisted of the cell layer covering the substrate (glass slide) and the mucosal pellicle at the cell surface. Mechanical tests were carried out on this model depending on MUC1 isoforms.

### Tribological measurement

The tribological approach used in this study is based on measuring the evolution of friction forces at the surface of the oral epithelium model. Tribological measurements were carried out using a homemade tribometer (Fig. 6a). The light load linear biotribometer consists of 2 sensors: a normal sensor (LSB200–20 g, Futek, USA) and a tangential piezoelectric sensor (9215 A, Kistler, Switzerland) which measure normal and tangential forces during the test between a spherical silicone indenter and the oral epithelium model, respectively. The spherical silicon indenter has a radius of curvature equal to $\:3 mm$ and a reduced Young’s modulus $\:{\text{E}}^{\text{*}}\sim150\text{k}\text{P}\text{a}$ measured by indentation. Furthermore, two piezoelectric tables (P-629.1CD, Physik Instrumente, Germany) ensured the normal and the tangential movements between the probe and the surface sample, as shown in Fig. 6a.b. This device was used to out friction tests at the micrometric scale and under light load to avoid damaging the cell layer. A normal load of $\:0.5mN$ was applied to the oral epithelium model with the indenter This set point was maintained and controlled using a PID regulator throughout the test to avoid the viscoelastic effects of the materials involved. Once the set point was reached, the tribological test started at a linear speed of $\:300\mu\:m/s$ and each test consisted of 10 cycles, as shown in Fig. 6c. Each experimental condition was repeated 8 times to ensure measurement repeatability. These values were chosen to avoid damaging the cell layer during the test and to ensure that the test was carried out on the cell layer and not on the substrate (see Table 6). During the tests, the normal and tangential forces were measured to characterize the lubricating properties of MP and MUC1 through the calculation of the friction coefficient and the energy dissipated by friction.

**Fig. 5:**
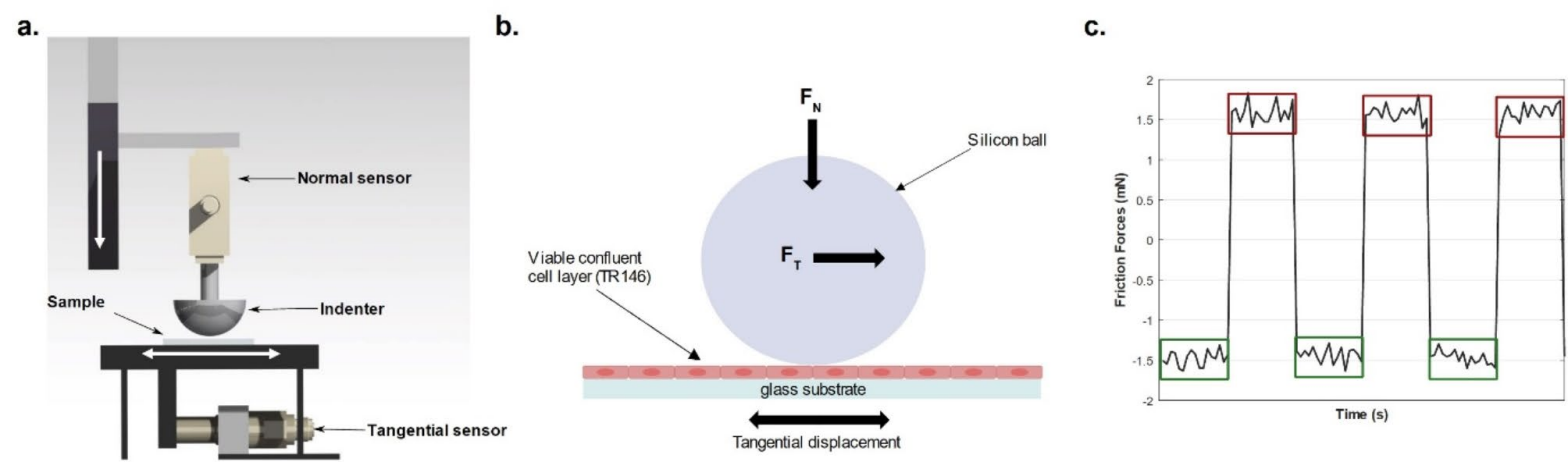
(**a**) Schematic representation of the in vitro bio-tribometer developed at LTDS as part of this project - This 3D model was created by the authors using the software Catia V5-6R2017 (licensed to École Centrale de Lyon, Dassault Systèmes, https://www.3ds.com/products-services/catia/), and a 3D rendering was performed to obtain this image. (**b**) Diagram of the contact during the tribological test between the indenter and the epithelium model. (**c**) Friction forces measured with the device during the reciprocating, sliding motion. Sliding areas represented by the green and red regions, are used to calculate the friction coefficient.

**Fig. 6:**
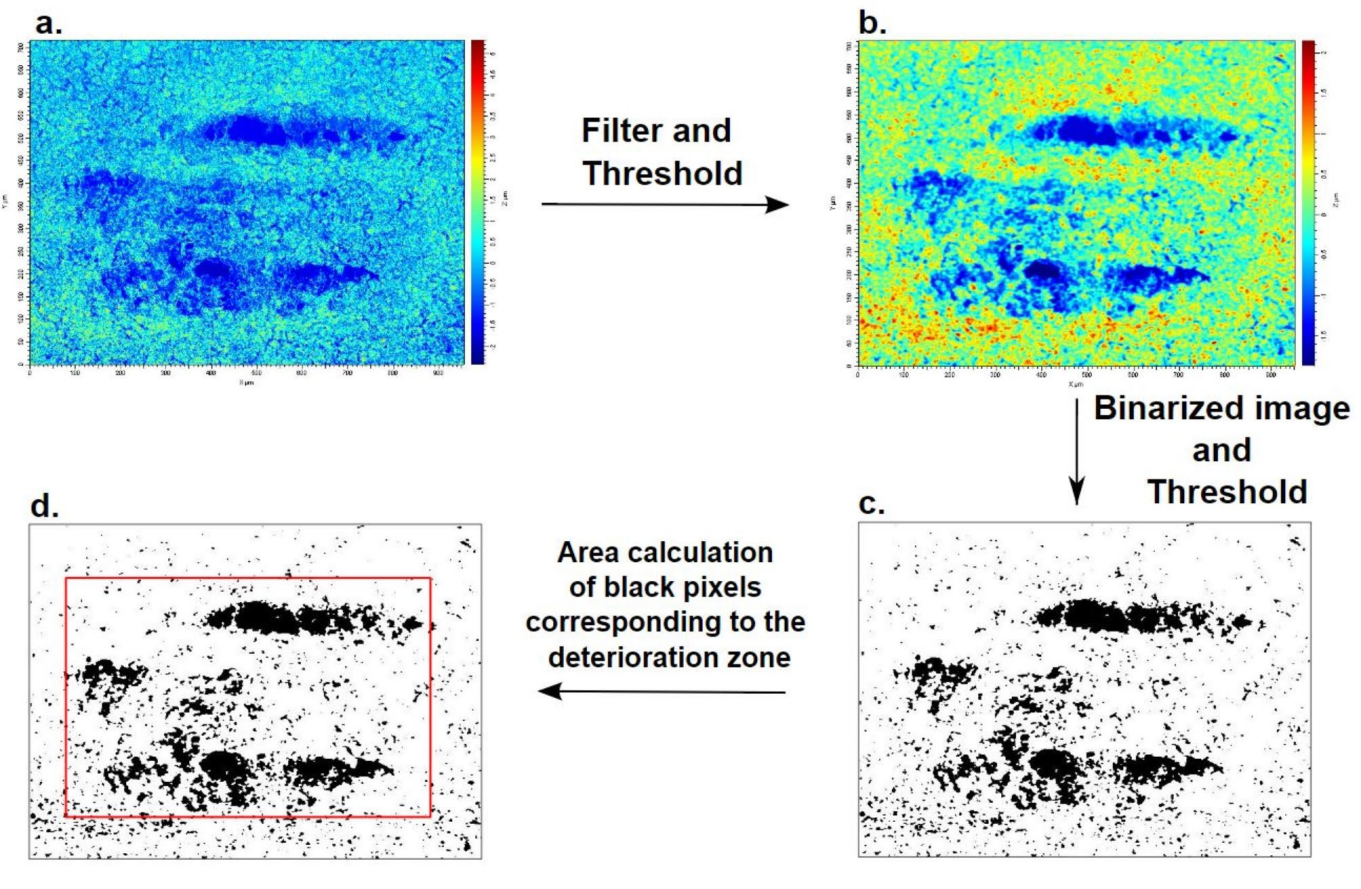
(**a**) An example of a raw image of a friction trace obtained on an oral epithelium model (MUC1/Y-LSP isoform) using optical interferometry. (**b**) The same image as in (**a**) after filtering and thresholding. (**c**) An image of the friction trace after binarization and thresholding, resulting in a black and white pixel image. (**d**) Calculation of the area of black pixels in the image, corresponding to the degraded areas.

**Table 6:** Experimental parameters of tribological tests.

Silicon ball radius	$\:1.5 mm$
Substrate	Glass side
Normal load speed	$\:1\mu\:m/s$
Imposed normal force	$\:0.5mN$
Friction stroke	$\:500\mu\:m$
Friction speed	$\:300\mu\:m/s$
Number of cycles	$\:10$
Contact threshold	$\:0.1mN$

#### Method

Once the tests were completed, the average tangential force cycle by cycle was calculated as follows:

$$\:{F}_{tmean}=\:\frac{{{F}_{Tmean}}_{max}+\:\left|{{F}_{Tmean}}_{min}\right|}{2}$$

One cycle corresponds to the probe’s movement to the end of the stroke and its return to the original point. The mean friction force for one cycle was calculated from the slip zones, where the friction force was constant, Fig. 6c. The average friction force was calculated over the slip zones (green and red rectangle). $\:{{F}_{Tmean}}_{max}$ represents the average tangential force on the red area, which corresponds to the forward motion. $\:{{F}_{Tmean}}_{min}$ corresponds to the average tangential force on the green area, representing the return motion.

Then, in a very conventional way, the friction coefficient, $\:\mu\:$ for each cycle was defined as:

$$\:\mu\:=\:\frac{{F}_{tmean}}{{F}_{Nmean}\:}$$

Where $\:{F}_{tmean}$ and $\:{F}_{Nmean}$ are respectively the mean tangential force during sliding and the mean normal force.

In this work, the energy dissipated by friction $\:{E}_{d\_cycle}$ (mJ) was also defined to characterizes the friction phenomena. This parameter was used to estimate the wear caused by friction, cycle by cycle. The dissipated energy was calculated using the area under the hysteresis formed during the cycle by plotting the tangential force as a function of the tangential displacement as follows:

$$\:{E}_{d\_cycle}={\int\:}_{-{\updelta\:}}^{{\updelta\:}}{F}_{T}\left({\updelta\:}\right)\hspace{0.17em}\text{d}{\updelta\:}$$

where $\:\delta\:$ is the linear displacement and $\:{F}_{T}$ is the tangential force during one cycle.

The cumulative energy dissipated, $\:Ed$ (mJ)? during the test was calculated as the sum of the dissipated energies cycle by cycle:

$$\:Ed={\sum\:}_{i=1}^{N}{E}_{d\_cycle}\left(i\right)$$

where N is the number of cycles.

### Damage analysis

To determine degradation following friction testing, the probe trace left on the epithelium model, Fig. 6a, was measured. Immediately after the friction test, the models of the oral epithelium were fixed using a 4% solution of Paraformaldehyde (PFA, Thermo Fisher Scientific, USA) in PBS (Thermo Fisher Scientific, USA) and dehydrated in four successive ethanol baths (70%, 80%, 90%, 100%). After complete drying, an image of the friction trace was captured by optical interferometry (Bruker, USA), Fig. 6a.

To quantify the deterioration, the open-source image processing software ImageJ was used. The images were first processed by a low-pass filter and then a threshold in order to limit the effects of noise and image artefacts, Fig. 6b. Finally, the images were binarized (0: Black and 255: White), using another threshold Fig. 6c. The area of the black pixels on the image corresponding to the degraded zone was measured, Fig. 6d. This region corresponds to the area of deterioration used in this work as a mechanical indicator of the state of lubrication.

### Statistical analysis

A statistical analysis was performed to ensure statistically significant differences. Firstly, a Kruskall-Wallis test was performed to study the overall similarity, providing an initial indication of the overall similarity between the groups. Then, Mann-Whitney tests were conducted, comparing each pair of samples pairwise. The objective was to determine if statistically significant differences existed between these specific groups. Also, in order to minimize errors in p-values caused by the repetition of tests, a Bonferroni correction was applied. These tests provided us with the p-value, indicating the level of confidence we could place in the results, with a risk level of $\:\alpha\:\le\:0.05$. The data analysis was performed using Matlab^®^. Significance codes: p = 0 < *** < 0.001 < ** < 0.01 < * < 0.05 <".".

## Conclusion

In conclusion, our study employing cellular models of oral mucosa based on the TR146 cell line expressing various isoforms of MUC1 provided valuable insight into the pivotal role played by MUC1 along with its VNTR and SEA domains, in influencing the lubricating properties of the mucosal pellicle and the intrinsic lubrication of the epithelium. These findings underscore the mucosal pellicle’s function as a lubricant, effectively mitigating mechanical forces acting upon the oral mucosa. Significantly, the presence of MUC1 emerges as a key factor enhancing the lubricating properties of the mucosal pellicle.

This enhancement is attributed to several factors. Firstly, it involves an increase in the number of bound salivary proteins, contributing to the formation of the mucosal pellicle in the presence of MUC1. Secondly, our results unequivocally demonstrate the collaborative roles of both the VNTR and SEA domains in augmenting the overall lubricating properties of the mucosal pellicle. Remarkably, the presence of the VNTR domain not only adds to the lubricating properties but also appears to synergize with salivary proteins, further enhancing the overall lubrication efficacy of the mucosal pellicle. In addition, the self-cleavage mechanism of the SEA domain, facilitating the disruption of the two MUC1 subunits, emerges as an efficient strategy for dissipating energy resulting from mechanical stress at the surface of the oral mucosa.

These cellular models of oral mucosa hold promise as valuable tools for investigating the multifaceted roles of MUC1 and the mucosal pellicle, not only in astringency but also in other aspects of flavour perception, such as aroma persistence. These findings deepen our understanding of the complex interplay between mucins, oral lubrication, and sensory experiences, opening new avenues for future research in the field.

## Data Availability

All data generated or analyzed during this study are included in this published article and its supplementary information files.
